# Enhancing transglutaminase production of *Streptomyces mobaraensis* by iterative mutagenesis breeding with atmospheric and room-temperature plasma (ARTP)

**DOI:** 10.1186/s40643-017-0168-2

**Published:** 2017-08-12

**Authors:** Ying Jiang, Yue-Peng Shang, Hao Li, Chao Zhang, Jiang Pan, Yun-Peng Bai, Chun-Xiu Li, Jian-He Xu

**Affiliations:** 1 State Key Laboratory of Bioreactor Engineering, East China University of Science and Technology, Shanghai, 200237 People’s Republic of China; 2 Shanghai Collaborative Innovation Center for Biomanufacturing Technology, East China University of Science and Technology, Shanghai, 200237 People’s Republic of China

**Keywords:** Atmospheric and room-temperature plasma, Breeding, *Streptomyces mobaraensis*, Transcription level, Transglutaminase

## Abstract

**Objectives:**

To improve the fermentation production of transglutaminase (TGase) from *Streptomyces mobaraensis* for applications in the food industry, the atmospheric and room-temperature plasma (ARTP) mutagenesis was applied to breed *S. mobaraensis* mutants with increased TGase production.

**Results:**

After eight rounds of iterative ARTP mutagenesis, four genetically stable mutants, *Sm*5-V1, *Sm*6-V13, *Sm*2-V10, and *Sm*7-V12, were identified, which showed increased TGase production by 27, 24, 24, and 19%, respectively. The best mutant *Sm*5-V1 exhibited a maximum TGase activity of 5.85 U/mL during flask fermentation. Compared to the wild-type strain, the transcription levels of the zymogen TGase genes in the mutants increased significantly as indicated by quantitative real-time PCR, while the gene nucleotide sequences of the mutants did not change at all. It was shown that the overexpression of TGase zymogen gene in the mutants contributes to the increase in TGase production.

**Conclusions:**

ARTP is a potentially efficient tool for microbial mutation breeding to bring some significant changes required for the industrial applications.

**Electronic supplementary material:**

The online version of this article (doi:10.1186/s40643-017-0168-2) contains supplementary material, which is available to authorized users.

## Background

Transglutaminases (TGase, EC 2.3.2.13), also referred to protein-glutamine γ-glutamyltransferases, are enzymes capable of catalyzing acyl-transfer reactions between the γ-carboxamide group of protein or peptide-bound glutamine and ε-amino group of lysine or other primary amines (Zhu et al. 1995; Martins et al. 2014). The covalent modifications of proteins promoted by TGase facilitate extensive applications in food, medicine, and other industries. TGases are widely distributed in prokaryotes and eukaryotes. TGases from animal tissues are Ca^2+^-dependent enzymes that lead to the precipitation of proteins from food containing casein, soybean globulin, or myosin (Martins et al. 2014). However, the scarcity of resources as well as complexity of further extraction and purification still limits their applications. Microbial transglutaminases (MTG) that were usually found in *Streptomyces* (Duran et al. 1998; Marx et al. 2008) and *Bacillus* (Kobayashi et al. 1998; Soares et al. 2003) species have a wider application due to their advantages as Ca^2+^-independent activity, thermostability, and broad specificity for acyl donors (Salis et al. 2015). Up to now, MTG for applications in the food processing were mainly produced by fermentation of *Streptomyces mobaraensis* (Yokoyama et al. 2004). TGase from *S. mobaraensis* was known to be secreted as a zymogen and then activated by proteolytic processing to the enzymatically active mature form (Zotzel et al. 2003a, b). Its pro-region is essential for efficient protein folding, secretion, and suppression of the enzymatic activity (Yurimoto et al. 2004). Considering the fact that genetically engineered strains are somehow restricted in the food industry, it is more feasible to improve TGase production by mutation breeding.

Microbial mutation breeding by altering the genomes shows great significance for biotechnology researches and applications (Tan et al. 2014; Kumar 2015). Recently, a novel and efficient mutation tool called atmospheric and room-temperature plasma (ARTP) has been successfully employed to cause some significant changes in enzyme activity, biochemical productivity, and metabolism without lethal damages (Guo et al. 2011; Lu et al. 2011; Xu et al. 2012; Wang et al. 2014). Compared to the conventional mutation breeding methods, ARTP mutagenesis shows some distinct advantages, such as low costs, low and controllable plasma temperatures, various active chemical species with a high density, rapid mutation, flexible and secure operations (Laroussi 2005; Zhang et al. 2014, 2015).

In this study, the iterative ARTP mutagenesis was applied to *S. mobaraensis* for breeding mutants with increased TGase production. Protocols for iterative rapid mutation of *S. mobaraensis* with helium-driven ARTP system and effective tube screening method of the mutants were established. The transcription levels and the nucleotide sequences of the gene (pro-*sm*tg) encoding TGase zymogen were compared between the mutants and the wild-type strain to explain the reasons for the enhanced TGase production, which may provide a valuable guidance for further researches on mechanisms regarding ARTP effects on the whole cells and the intracellular bio-macromolecules.

## Methods

### Materials

l-glutamic acid γ-monohydroxamate was purchased from Sigma-Aldrich Co., Ltd. (Shanghai, China). *N*-α-Carbobenzoxy-l-glutaminyl-glycine (*N*α-CBZ-Gln-Gly) was purchased from Civi Chemical Technology Co., Ltd. (Shanghai, China). Reduced glutathione was purchased from Aladdin Reagent Co., Ltd. (Shanghai, China). All-in-One First-Strand cDNA Synthesis SuperMix (One-Step gDNA Removal) and *TransStart* Tip Green qPCR SuperMix that were used for RT-PCR (Reverse Transcription PCR) and qRT-PCR (quantitative Real-Time PCR) were purchased from TransBionovo Co., Ltd. (Beijing, China). All other reagents were obtained from commercial sources and were of analytical grade.

### Strains and media

*Escherichia coli* DH5α was used as the host strain for the recombinant DNA manipulations. *Streptomyces mobaraensis*, the wild-type strain (*S. mobaraensis* ECU7480, stored in our lab) and the mutants generated by ARTP mutagenesis, were grown on a solid medium comprising 20 g/L soluble starch, 3 g/L tryptone, 1 g/L KNO_3_, 0.5 g/L K_2_HPO_4_·3H_2_O, 0.5 g/L MgSO_4_·7H_2_O, 0.5 g/L NaCl, 0.01 g/L FeSO_4_·7H_2_O, and 1.5–2.0% (w/v) agar at pH 7.4–7.6 and 30 °C for 5 days. Both tube and flask fermentations were conducted using a seeding medium (20 g/L glycerol, 20 g/L tryptone, 5 g/L yeast extract, 2 g/L MgSO_4_·7H_2_O, 2.62 g/L K_2_HPO_4_·3H_2_O, 2 g/L KH_2_PO_4_ , pH 7.0) at 30 °C for 24 h to prepare the inoculums and a fermentation medium (20 g/L glycerol, 20 g/L tryptone, 5.5 g/L corn steep powder, 5 g/L yeast extract, 2 g/L MgSO_4_, 2.62 g/L K_2_HPO_4_·3H_2_O, 10 g/L CaCO_3_ , pH 7.2) at 30 °C with 8–10% inoculum size for the production of TGase.

### Procedures for iterative mutagenesis with ARTP and directed screening

**Fig. 1:**
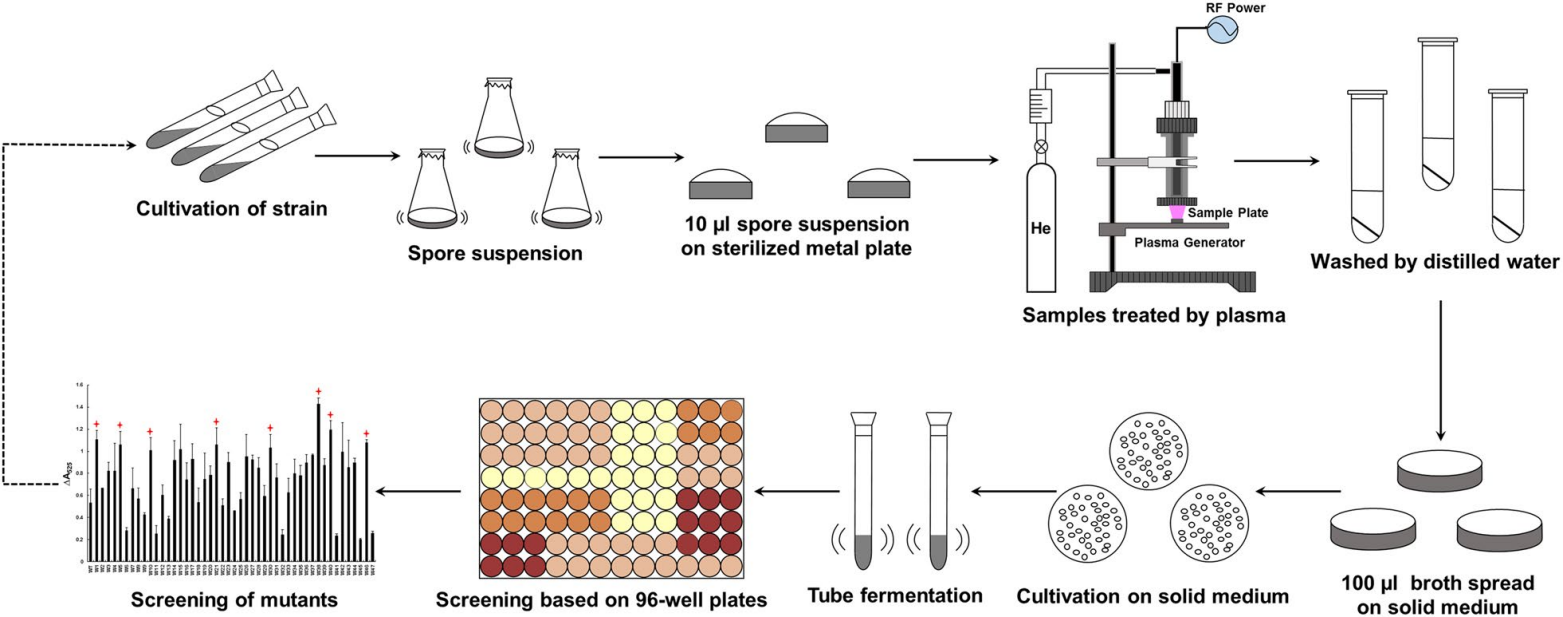
Scheme of ARTP mutagenesis for microbial breeding

The procedures for iterative mutation of *S. mobaraensis* genome with ARTP mutation breeding system purchased from Si Qing Yuan Biotechnology Co., Ltd. (Beijing, China) and the following screening are shown in Fig. 1. In this study, pure helium was used as the working gas at a flow rate of 10 slpm (standard liters per minute). The RF power input was 40 W. The distance (*D*) between the plasma torch nozzle exit and the sample plate was 4 mm and the plasma jet temperature was below 30 °C. To provide different dosages of the active species in the plasma jet region, 10 μL of the spore suspension was pipetted onto the stainless minidisk and then exposed to ARTP jet for different treatment times ranging from 10 to 60 s. Spores without treatment were used as the control.

After ARTP mutation, the spore suspension was transferred onto solid medium. Single colonies from the solid medium were selected randomly and inoculated into a test tube containing 3 mL fermentation medium for 96 h fermentation. The cell-free supernatant of fermentation broth was withdrawn for TGase activity detection. The top eight mutants with increased TGase production were chosen as the starting strains for the next round of ARTP mutagenesis. After eight rounds of iterative ARTP mutagenesis, flask cultivation was performed to verify the TGase production of selected mutants from shake tube screening. The selected mutants grown on the solid medium were cultivated in 250-mL flasks containing 25 mL seeding medium at 30 °C for 24 h. Then, 2.5 mL of the seeding culture was transferred into 250-mL shake flasks containing 25 mL fermentation medium and cultured at 30 °C. Aliquots of the cell-free supernatant were taken at different time intervals for examination of the TGase activity. Additionally, the genetic stability of the identified mutants was evaluated by eight rounds of subcultures. The TGase production of the mutants was examined by shake flask fermentation in every two subcultures.

### Evaluation of ARTP mutagenesis of *S. mobaraensis*

The lethal rates of the spores under different treatment times were evaluated according to Eq. 1. The mutation rate and the positive mutation rate were calculated based on Eqs. 2 and 3, respectively.

1 $${\text{Lethal rate }}\left( \% \right) \, = \frac{U - T}{U} \times 100$$

2 $${\text{Mutation rate }}\left( {R_{\text{M}} } \right) \, \left( \% \right) \, = \frac{M}{T} \times 100$$

3 $${\text{Positive mutation rate }}\left( {R_{\text{P}} } \right) \, \left( \% \right) \, = \frac{P}{M} \times 100,$$

where *U* is the total colony count of the sample without treatment, *T* is the total colony count after treatment with ARTP, *M* is the total colony count of mutants with different TGase production from the wild-type strain, and *P* is the total colony count of mutants with increased TGase production than that of the wild-type strain. All the colony numbers were obtained by the colony forming unit (CFU) method on a solid medium.

### Activity assay of TGase

The activity of TGase was measured according to the colorimetric hydroxamate procedure (Grossowicz et al. 1950) using *N*α-CBZ-Gln-Gly as the substrate. The calibration curve was prepared using l-glutamic acid γ-monohydroxamic acid as a standard. One unit (U) of TGase activity was defined as the amount of enzyme that catalyzes the formation of 1 μmol l-glutamic acid γ-monohydroxamate per minute at 37 °C.

### Quantitative real-time PCR analysis

Total RNAs of *S. mobaraensis* ECU7480 and its mutants were extracted after cultivation for different times. The mycelium pellets were collected by centrifugation and then frozen in liquid nitrogen immediately. The extraction of total RNAs was performed using the SV Total RNA Isolation System (Promega, USA) according to the manufacturer’s protocol. The quantity and quality of the isolated RNAs were examined by NanoDrop 2000c UV–Vis spectrophotometer (Thermo Scientific, USA) and agarose gel electrophoresis. Subsequently, the reverse transcription PCR (RT-PCR) was carried out using 1 μg total RNA as the template. The gene transcription level of pro-*sm*tg during fermentation process was investigated by quantitative real-time PCR (qRT-PCR) with primers listed in Table 1. The qRT-PCR reaction consisted of an initial denaturation at 94 °C and 40 amplifications cycles of 5 s at 94 °C and 60 s at 64 °C. The target gene transcription level was normalized internally to that of 16S rRNA gene for its transcription during overall growth stages was relatively stable.

**Table 1:** Primers used in this study

Name	Sequences (5′–3′)	Application
16S rRNA_L1	AGCAGCGGAGCATGTGGCTT	16S rRNA gene transcription
16S rRNA_R1	TGCGCTCGTTGCGGGACTTA	
pro-*sm*tg-L1	CATGTCGAGGGACAGGAACA	pro-*sm*tg gene transcription
pro-*sm*tg-R1	TTGCGGAACTTGCTCTCGTA	
pro-*sm*tg-FP	CGGAATTCATGCCGTCCGCAGGC	pro-*sm*tg gene amplification
pro-*sm*tg-RP	CCCAAGCTTTCACGGCCAGCCCTG	

### Cloning of pro-*sm*tg gene from *S. mobaraensis* mutants

Genomic DNAs were obtained from *S. mobaraensis* mutants and the wild-type strain. The primers used for the PCR are listed in Table 1. The PCR procedure was set as follows: (95 °C, 5 min) 1 cycle; (94 °C, 1 min; 65 °C, 30 s; 72 °C, 90 s) 30 cycles; and (72 °C, 10 min) 1 cycle. The amplified PCR products (1200 bp) were ligated into plasmid pMD-19T and sequenced for alignment to that of the wild-type strain.

## Results and discussion

### Mutation and screening of the mutants

**Fig. 2:**
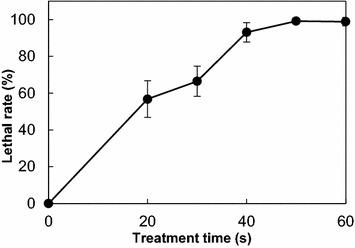
Lethal rate of *S. mobaraensis* by ARTP mutagenesis

A well-controlled lethal rate is fundamental for effective mutation and screening of the mutants. The lethal rates of *S. mobaraensis* with respect to various treatment times are shown in Fig. 2, which indicated that the lethal rate increased to 66.5, 93.1, and 99.2%, respectively, after treated for 30, 40, and 50 s. When the samples were treated for 50 s or even longer, no spores could survive. According to previous reports (Guo et al. 2011; Hua et al. 2010), a lethal rate of 90% was considered appropriate. Besides, keeping the lethal rate high is necessary for the effective mutation and selection of mutant strains. Therefore, the ARTP treatment time applied in this study was determined as 40 s.

**Fig. 3:**
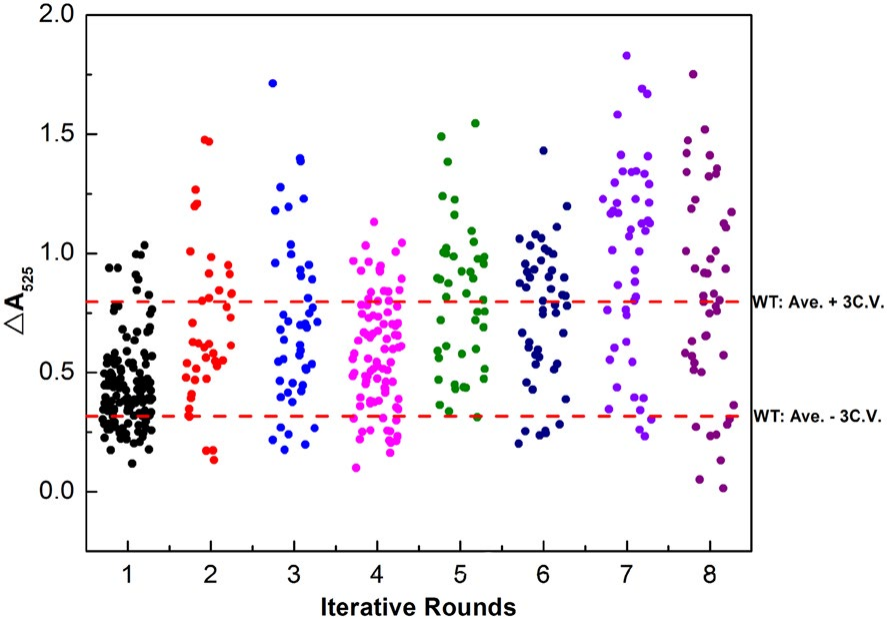
Distribution of screening results for each round of ARTP mutagenesis. $\Delta A_{525} = A_{525, Sample} - A_{525, contorl}$, which is proportional to the TGase activity

After eight rounds of iterative ARTP mutagenesis, 501 mutants in total were selected for tube-fermentation screening, as shown in Additional file 1: Figure S1. To investigate the accumulative effect on TGase production by iterative ARTP mutagenesis, the mutation and screening results for each round were collected and compared. The statistic results shown in Table 2 and Fig. 3 indicated that the proportion of positive mutants was scaled up with the increase of iterative rounds. Moreover, the TGase production of the best mutant for each round presented a fluctuant improving trend. Thus, the increase of iterative rounds might exhibit an accumulative effect on TGase production of the mutants.

**Table 2:** Mutation and screening results for each round of ARTP mutagenesis

Iterative round	*R*_M_ (%)	*R*_P_ (%)	Average $\Delta A_{525}$ of mutants
WT	–	–	0.56 ± 0.08
1	32.1	6.4	0.44 ± 0.19
2	48.8	36.6	0.69 ± 0.33
3	45.7	32.6	0.71 ± 0.35
4	34.8	19.1	0.58 ± 0.23
5	56.8	54.6	0.81 ± 0.31
6	62.5	50.0	0.75 ± 0.28
7	74.5	68.1	0.98 ± 0.41
8	69.6	52.2	0.83 ± 0.43

### TGase production of the mutants

Shake flask fermentation was conducted to verify the potentially positive mutants identified in the tube fermentation. Finally, four mutants, *Sm*5-V1, *Sm*6-V13, *Sm*2-V10, and *Sm*7-V12, were identified in the ARTP breeding process, and the TGase production of these mutants is shown in Table 3. The highest TGase production reached 5.85 U/mL, which represented a 27% increase as compared with the wild-type strain.

**Table 3:** TGase production of *S. mobaraensis* wild-type and ARTP mutants

Strain	TGase titer (U/mL)	Relative production (%)
WT	4.60 ± 0.17	100 ± 4
*Sm*5-V1	5.85 ± 0.21	127 ± 5
*Sm*6-V13	5.72 ± 0.16	124 ± 4
*Sm*2-V10	5.68 ± 0.19	124 ± 4
*Sm*7-V12	5.49 ± 0.13	119 ± 3

The genetic stability is one of the key performance factors for a promising industrial strain because it reflects the potential mutations at the gene level (Ren et al. 2017). The genetic stability test results shown in Additional file 1:Figure S2 indicated that the TGase production of the identified mutants still remained stable after eight rounds of subcultures. These results proved that ARTP mutagenesis is a promising mutation breeding tool for industrial applications.

### Pro-*sm*tg gene expression level of the wild-type and mutant strains

The enhanced TGase production caused by ARTP mutagenesis may be attributed to two effects. A direct effect will happen if the structural gene (pro-*sm*tg) of TGase zymogen is altered, leading to the change in the specific activity of the protein. Indirect effect happens when the relevant genes regulating the expression of TGase zymogen are altered.

**Fig. 4:**
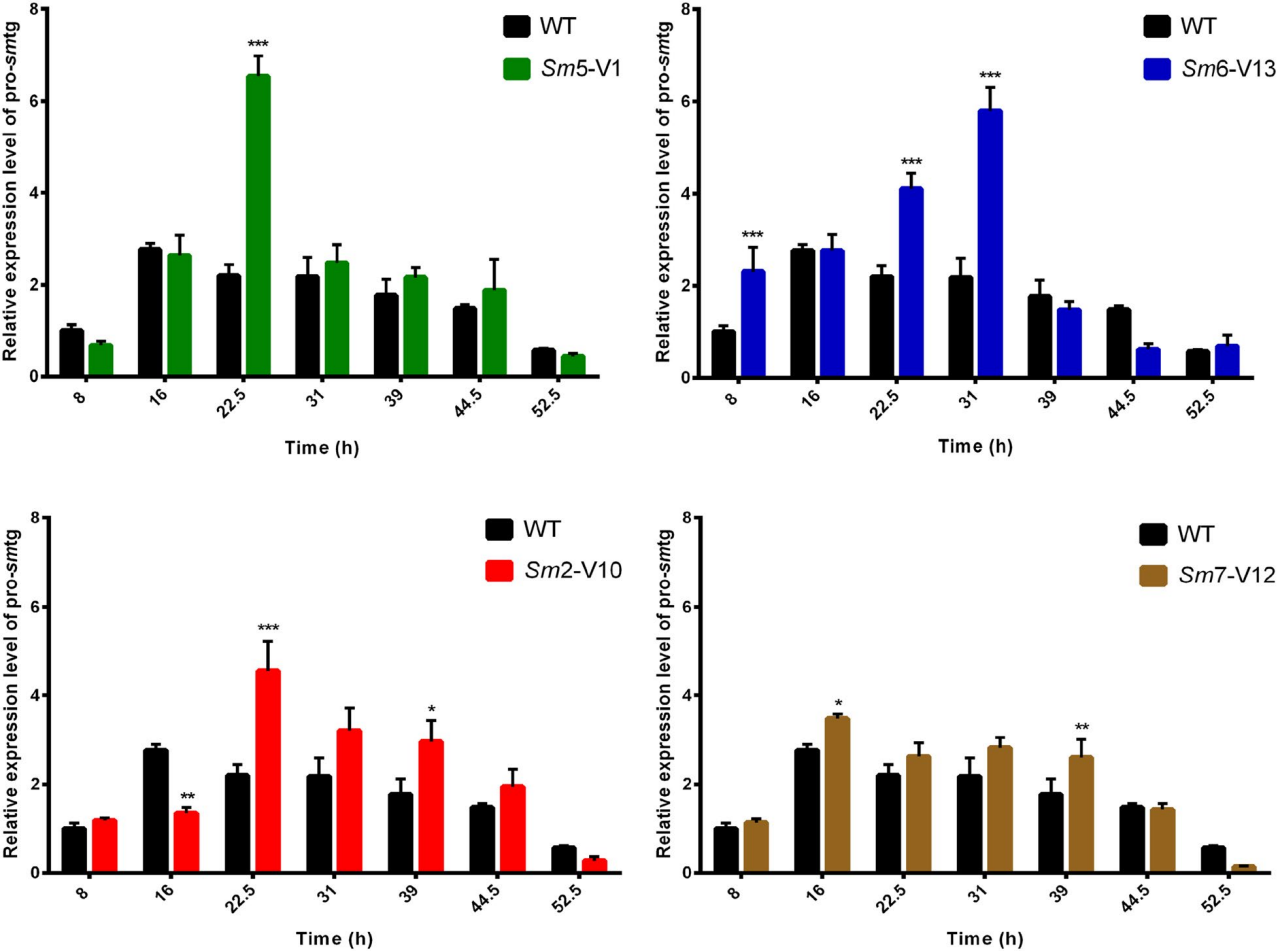
Transcription analysis of pro-*sm*tg gene by qRT-PCR. The pro-*sm*tg transcription levels were detected after flask fermentation for different times in wild-type strain (WT) and mutants *Sm*5-V1, *Sm*6-V13, *Sm*2-V10, and *Sm*7-V12, which were normalized to that of 16S rRNA gene by the $2^{ - \Delta \Delta Ct}$ method. The experiments were performed in three replications

Firstly, the pro-*sm*tg gene nucleotide sequences of the four mutants showed no differences from the original sequence of the native enzyme from the wild-type strain, as indicated by gene cloning and sequence alignment shown in Additional file 1: Figure S3. However, qRT-PCR results shown in Fig. 4 indicated that the pro-*sm*tg expression levels of the four mutants were remarkably improved as compared to that of the parental strain, which might lead to the increase in TGase production. The analysis from transcription level conducted in this study is of great importance for further investigation of metabolic changes caused by ARTP mutagenesis.

## Conclusions

Iterative ARTP mutagenesis was applied to improve the TGase production in *S. mobaraensis* as a novel and efficient mutation tool. As a result, four mutants, *Sm*5-V1, *Sm*6-V13, *Sm*2-V10, and *Sm*7-V12, with increased TGase production by 27, 24, 24, and 19%, respectively, were identified after eight rounds of iterative mutagenesis. The best mutant *Sm*5-V1 showed an enhanced TGase production of 5.85 U/mL. In addition, the results of sequence alignment and qRT-PCR revealed that the expression of TGase zymogen gene increased obviously under the ARTP treatment, while the gene sequence remained unchanged. The analysis of the target gene sequence as well as transcription level conducted in this study would provide a valuable guidance for further researches and applications of ARTP mutagenesis.

## Additional file

**Additional file 1.** Additional information.

## Abbreviations

ARTP: atmospheric and room-temperature plasma
TGase: transglutaminase
MTG: microbial transglutaminases
RT-PCR: reverse transcription PCR
qRT-PCR: quantitative real-time PCR
