# Supplementary material for: Enhancing transglutaminase production of Streptomyces mobaraensis by iterative mutagenesis breeding with atmospheric and room-temperature plasma (ARTP)
Source: Bioresour Bioprocess. 2017 Aug 12;4(1):37. doi: 10.1186/s40643-017-0168-2 (PMC5554476; doi:10.1186/s40643-017-0168-2)
Supplement: Supplementary file 1 — Additional file 1. Additional information. [file 40643_2017_168_MOESM1_ESM.docx]

Submitted to: ***Bioresources and Bioprocessing***

**Enhancing Transglutaminase Production of *Streptomyces mobaraensis* by Iterative Mutagenesis Breeding with Atmospheric and Room Temperature Plasma (ARTP)**

Ying Jiang^1^, Yuepeng Shang^1^, Hao Li^1^, Chao Zhang^1^, Jiang Pan^1^, Yunpeng Bai^1^, Chunxiu Li^1,*^, Jianhe Xu^1,2,*^

^1^ State Key Laboratory of Bioreactor Engineering and ^2^ Shanghai Collaborative Innovation Center for Biomanufacturing Technology, East China University of Science and Technology, Shanghai 200237, P. R. China

*Corresponding authors. Tel.: +86-21-6425-2498; Fax: +86-21-6425-0840; E-mails:

[chunxiuli@ecust.edu.cn](mailto:chunxiuli@ecust.edu.cn) (C.X. Li); jianhexu@ecust.edu.cn (J.H. Xu).

**Figure S1** Screening results of 501 *S. mobaraensis* mutants by tube fermentation.

(a) (b)

(c) (d)

**Figure S2** TGase production by flask fermentation during generations of *S. mobaraensis* mutants. (a), (b), (c), (d) represent the results of *Sm*5-V1, *Sm*6-V13, *Sm*2-V10 and *Sm*7-V12, respectively.

**Figure S3** Multiple alignment results of TGase zymogen amino acid sequences among the wild type and the four mutants.
